# Comparison of the clinical application values of PCT, hs-CRP and SAA detection in the early diagnosis of sepsis

**DOI:** 10.12669/pjms.36.7.2544

**Published:** 2020

**Authors:** Yuan-da Sui, Wei-na Xin, Lin-lin Feng

**Affiliations:** 1 Yuan-da Sui, Department of Critical Medicine, Liaocheng People’s Hospital, 252000, Liaocheng, Shandong, P. R. China; 2 Wei-na Xin, Department of Respiratory Medicine, Liaocheng People’s Hospital, 252000, Liaocheng, Shandong, P. R. China; 3 Lin-lin Feng, Department of Respiratory Medicine, Liaocheng People’s Hospital, 252000, Liaocheng, Shandong, P. R. China

**Keywords:** High-sensitivity C-reactive protein, Procalcitonin, Sepsis, Serum amyloid A

## Abstract

**Objectives:**

To investigate the clinical application values of procalcitonin (PCT), high-sensitivity C-reactive protein (hs-CRP) and serum amyloid A (SAA) in the early diagnosis of sepsis.

**Methods:**

In this retrospective analysis, 36 patients admitted to Liaocheng People’s Hospital were selected from May 2018 to July 2019. According to infectious disease diagnostic criteria, 17 patients were confirmed to have sepsis (observation group), and 19 patients were determined to be nonseptic (control group). The levels of PCT, CRP and SAA of patients were detected on admission, and the clinical application values of PCT, CRP and SAA for sepsis were compared.

**Results:**

Seventeen patients were included in the observation group, including 9 males and 8 females, with an average age of 52.18 ± 9.49 years; 19 patients were included in the control group, including 12 males and 7 females, with an average age of 51.53 ± 8.50 years. On admission, there were significant differences in white blood cell (WBC) count (*t* = 5.134), neutrophil count (*t* = 3.143), lymphocyte count (*t* = 2.510), PCT (*t* = 9.250), hs-CRP (*t* = 2.947) and SAA (*t* = 11.360) between the observation group and the control group, and the differences were statistically significant. For the comparison of clinical application values: the sensitivity of PCT, hs-CRP and SAA was 78.95%, 52.17% and 50.00%, respectively; the specificity of PCT, hs-CRP and SAA was 88.24%, 61.54% and 37.50%, respectively; the area under the ROC curve (AUC) of PCT, hs-CRP and SAA was 0.920, 0.684 and 0.870, respectively; the logistic regression coefficient of PCT, hs-CRP and SAA was -0.577, -0.028 and -0.009, respectively; and the 95% confidence interval (CI) of PCT, hs-CRP and SAA was 0.779-0.985, 0.508-0.828 and 0.716-0.958, respectively.

**Conclusion:**

Compared with hs-CRP and SAA, PCT had a higher clinical application value for sepsis, and PCT could be used as a reliable index for the early diagnosis of sepsis.

## INTRODUCTION

Sepsis is a systemic inflammatory response syndrome caused by infections that can result in multiple organ dysfunction and/or circulatory disturbance in severe cases. Moreover, severe trauma, burn, shock, infection and major surgery are the common inducing factors of sepsis. Sepsis is associated with a very high mortality and is the main cause of death of non-heart disease patients in the ICU.^1^ Therefore, timely diagnosis of sepsis to guide treatment has become an important task for clinicians. Laboratory tests on sepsis provided strong evidence for the diagnosis of sepsis, mainly including hematological indicators and pathogen culture. However, the culture and identification of pathogens is usually a time-consuming process; thus, changes in hematology related indicators play an increasingly important role in the diagnosis of sepsis. In this study, the levels of procalcitonin (PCT), high-sensitivity C-reactive protein (hs-CRP), serum amyloid A (SAA) and other inflammatory factors were detected in septic patients and nonseptic patients, and the clinical application values of PCT, hs-CRP and SAA in the diagnosis of sepsis were compared.

## METHODS

### Ethical approval

The study was approved by the Institutional Ethics Committee of Liaocheng People’s Hospital on March 15, 2020(No.2020020), *(Since project was not completed in time, it had to be renewed annually)* and written informed consent was obtained from all participants.

### Baseline information

From May 2018 to July 2019, 36 patients with suspected symptoms of sepsis (such as respiratory rhythm or frequency disorder, a disturbance of consciousness, high fever) admitted to Liaocheng People’s Hospital were selected. After confirmation by blood culture and clinical symptoms, 17 patients with sepsis were included in the observation group, including 9 cases of Gram-negative bacterial infection and 8 cases of Gram-positive bacterial infection, and 19 nonseptic patients were included in the control group.

### Specimen collection

On the next day after admission, peripheral venous blood (approximately 4 ml) was drawn from each patient in the state of fasting in the morning and placed in a heparin anticoagulation tube. Before testing, blood samples were centrifuged at a speed of 3500 r/minute, and the hematological test was carried out after centrifugation for 10 minute. Blood culture: venous blood samples of patients were extracted and placed in blood culture-specific bottles (10 ml per bottle), and blood culture-specific bottles were placed in a blood culture instrument.

### PCT detection

Electrochemical luminescence method was adopted with a detection threshold set at 2 ng/ml, and the PCT was detected using a Roche E170 analyzer.

### CRP detection

CRP was detected by a rate immunonephelometric assay, and the detection instrument was a Beckman IMMAGE.

### SAA detection

SAA was detected by a fixed-time immunonephelometric assay, and the detection instrument was a Siemens BN II.

## RESULTS

There were 17 patients included in the observation group (nine males and eight females), including nine cases of Gram-negative bacterial infection and eight cases of Gram-positive bacterial infection, with an average age of 52.18 ± 9.49 years. On admission, the WBC count was 15.21 ± 3.42 × 10^9^/L, the neutrophil count was 10.21 ± 1.45 × 10^9^/L, the lymphocyte count was 1. 00 ± 0.09 × 10^9^/L, the PCT was 8.43 ± 2.44 ng/ml, the hs-CRP was 90.28 ± 25.56 mg/L and the SAA was 464.96 ± 93.26 mg/L. There were 19 patients in the control group (12 males and 7 females), with an average age of 51.53 ± 8.50 years. On admission, the WBC count was 10.29 ± 2.27 × 10^9^/L, the neutrophil count was 8.90 ± 1.04 × 10^9^/L, the lymphocyte count was 0.91 ± 0.09 × 10^9^/L, the PCT was 2.43 ± 1.15 ng/mL, the hs-CRP was 70.64 ± 10.67 mg/L and the SAA was 191.97 ± 35.35 mg/L.

On admission, there were no significant difference in sex (*P* = 0.736) or age (*t* = 0.217) between the observation group and the control group. There were significant differences in WBC count (*t* = 5.134), neutrophil count (*t* = 3.143), lymphocyte count (*t* = 2.510), PCT (*t* = 9.250), hs-CRP 9 (*t* = 2.947) and SAA (*t* = 11.360) between the observation group and the control group, and the differences were statistically significant (Table-I and Table-II).

**Table-I:** Clinical baseline data of patients in the observation group and the control group

	Sex (male/female)	Age (years)	WBC count (×10^9^/L)	Neutrophil count (×10^9^/L)	Lymphocyte count (×10^9^/L)
Observation group (n=17)	9/8	52.18 ± 9.49	15.21 ± 3.42	10.21 ± 1.45	1.00 ± 0.09
Control group (n=19)	12/7	51.53 ± 8.50	10.29 ± 2.27	8.90 ± 1.04	0.91 ± 0.09
t		0.217	5.134	3.143	2.510
P	0.736^a^	0.830	< 0.001	0.003	0.017

*Note:* ^a^, Fisher exact probability; WBC, white blood cell

**Table-II:** Laboratory detection levels of PCT, hs-CRP and SAA of patients in the observation group and the control group.

	PCT (ng/mL)	hs-CRP (mg/L)	SAA (mg/L)
Observation group (n=17)	8.43 ± 2.44	90.28 ± 25.56	464.96 ± 93.26
Control group (n=19)	2.43 ± 1.15	70.64 ± 10.67	191.97 ± 35.35
t	9.250	2.947	11.360
P	< 0.001	0.008	< 0.001

*Note:* PCT, procalcitonin; hs-CRP, high-sensitivity C-reactive protein; SAA, serum amyloid A

**Table-III:** Comparison of clinical application values among PCT, hs-CRP and SAA.

	Sensitivity (%)	Specificity (%)	AUC	Logistic regression coefficient	95% CI
PCT	78.95	88.24	0.920	-0.577	0.779-0.985
hs-CRP	52.17	61.54	0.684	-0.028	0.508-0.828
SAA	50.00	37.50	0.870	-0.009	0.716-0.958

*Note:* PCT, procalcitonin; hs-CRP, high-sensitivity C-reactive protein; SAA, serum amyloid A; AUC, area under the receiver operating characteristic (ROC) curve; CI, confidence interval

Comparison of clinical application values (Table-II):

***PCT:*** sensitivity 78.95%, specificity 88.24%, AUC 0.920, logistic regression coefficient -0.577, and 95% CI 0.779-0.985;

***CRP:*** sensitivity 52.17%, specificity 61.54%, AUC 0.684, logistic regression coefficient -0.028, and 95% CI 0.508-0.828;

***SAA:*** sensitivity 50.00%, specificity 37.50%, AUC 0.870, logistic regression coefficient -0.009, and 95% CI 0.716-0.958.

## DISCUSSION

Sepsis is a common cause of death for inpatients in the ICU, and it often begins with infection. In the early stage, sepsis may manifest as physical discomfort, fever and other nonspecific symptoms, and the physical signs were changeful and diversified; thus, it is difficult to make a clear diagnosis.^1^,^2^ Common high-risk factors included the following:

1. Age: too old (>75) or too young.
2. Recent history of surgery or childbirth/abortion.
3. Body wounds or skin ruptures.
4. Poor immune function.
5. History of diabetes and other chronic diseases, which may lead to damage of skin barrier function.^3^,^4^

In clinical work, the diagnosis of sepsis was realized by carrying out laboratory tests on patients’ blood samples to guide the follow-up treatment, and the prognosis of sepsis could also be determined by detecting levels of various factors. The levels of PCT, CRP and SAA have important clinical significance in the diagnosis and treatment of Sepsis and other infectious diseases.^5^-^10^

**Fig. 1:**
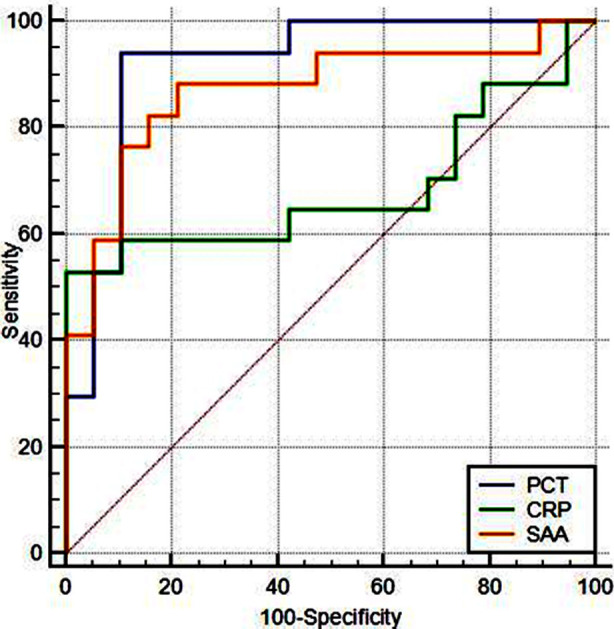
ROC curves of PCT, hs-CRP and SAA. ***Note:*** PCT, procalcitonin; hs-CRP, high-sensitivity C-reactive protein, SAA, serum amyloid A; ROC, receiver operating characteristic.

SAA is one of the markers of inflammatory activity in the acute response phase, mainly produced by macrophages.^11^ SAA has the characteristics of a low basal level and high induced expression, and it is produced in large quantities in the inflammatory acute response period.^12^ Some studies have shown that recombinant human serum amyloid A (rhSAA) had chemotactic effects on phagocytes and had effects in prolonging the life of neutrophils and inducing the expression of IL-1, IL-10 and other cytokines.^13^-^15^

CRP, an acute-phase protein mainly produced by the liver, plays an important role in the inflammatory process and the body infection response process.^16^ In addition, CRP often acts as an inflammation biomarker, and CRP can increase exponentially to a level 1000 times the normal level in bacterial infections.^17^ As an important marker in the process of inflammation, CRP can also induce increased secretion of IL-6, IL-1 and other cytokines during infection.^14^ At the same time, CRP can activate complement pathways and induce apoptosis to inhibit macrophage-driven pro-inflammatory responses.^18^,^19^ Szalai^20^ and other scholars found that CRP could promote the early clearance of *Salmonella enterica* serovar typhimurium in the blood, thus reducing the transmission to the liver and further increasing the resistance of mice to bacterial infections in a mouse model. Moreover, a previous study suggested that CRP could mediate the host’s response to *Staphylococcus aureus*, including the protective function against infections and the role in increasing phagocytosis of pathogens^.21^

PCT is encoded by the calcitonin I (CALC-1) gene on chromosome 11, containing 114-116 amino acids. PCT is almost entirely produced in the thyroid glands, and it is converted to calcitonin before entering the systemic circulation. Additionally, PCT was often used as a biomarker to distinguish the presence of bacterial infections. Specifically, the serum level of PCT in the human body is 0.1 ng/ml or below, while 0.25 ng/ml or above may indicate the presence of bacterial infections. In patients with systemic bacterial infections, the production of PCT was promoted in spleen, pancreas, kidney and other tissues under the induction of bacteria and pro-inflammatory factors, so that PCT could enter the whole body.^22^ It has been demonstrated that the PCT level could be used to guide clinical treatment, and shortened length of stay and reduced mortality were realized.^23^ However, it should be noted that increased PCT levels were also observed in patients with trauma or surgery, thus leading to false-positive results.^24^ Furthermore, PCT had a shorter half-life than CRP, and PCT showed a faster increase in bacterial infections. In general, PCT increased rapidly within 2-3 hours, peaked within 6-8 hours, lasted for 12-48 hours, and decreased rapidly in 2-3 days after infection control, while CRP increased significantly after 12 hours of inflammation. The abovementioned characteristics allowed doctors to diagnose sepsis earlier and monitor the progress better.^25^-^27^ Some studies have revealed that as a biomarker, PCT had better sensitivity and specificity than CRP and IL-6 in the diagnosis of sepsis.

### Limitations of the study

At the same time, this study also has some limitations, such as the number of cases is relatively short.

## CONCLUSION

The study found that: PCT was superior to hs-CRP and SAA with respect to sensitivity and specificity in the early diagnosis of sepsis. In addition, the results of our study also suggested that PCT had the best logistic regression coefficient and area under the ROC curve (AUC), indicating that PCT had higher clinical application value than hs-CRP and SAA in the early diagnosis of sepsis.

### Authors’ Contributions:

**YDS** designed this study and prepared this manuscript and is responsible and accountable for the accuracy and integrity of the work.

**LLF** collected and analyzed clinical data.

**WNX** significantly revised this manuscript.
